# The Dutch practice of euthanasia and assisted suicide in patients suffering from psychiatric disorders: a qualitative case review study

**DOI:** 10.3389/fpsyt.2024.1452835

**Published:** 2024-10-28

**Authors:** Fenne Bosma, Kelly Romana Mink, Johannes Jozef Marten van Delden, Agnes van der Heide, Suzanne van de Vathorst, Ghislaine Jose Madeleine Wilhelmien van Thiel

**Affiliations:** ^1^ Department of Public Health, Erasmus Medical Center Rotterdam, Rotterdam, Netherlands; ^2^ Department of Bioethics and Health Humanities, Julius Center for Health Sciences and Primary Care, University Medical Center Utrecht, Utrecht, Netherlands; ^3^ Department of Medical Ethics, Philosophy, and History of Medicine, Erasmus Medical Center Rotterdam, Rotterdam, Netherlands

**Keywords:** physician-assisted suicide, psychiatric disorder, qualitative study, euthanasia, case review

## Abstract

**Importance:**

Euthanasia or assisted suicide (EAS) in patients suffering from a psychiatric disorder (PD) is a controversial topic worldwide. In the Netherlands, this practice is regulated by law. All cases of EAS have to be reported and are assessed by the Regional Euthanasia Review Committees (RTEs), who publish a selection of all cases on their website.

**Objective:**

To provide insight into the Dutch practice of EAS in patients suffering from a psychiatric disorder.

**Design, setting and participants:**

We performed a retrospective case review study in which all published cases of EAS in patients suffering from a PD between 2017 and 2022 were analyzed.

**Intervention(s) or exposure(s):**

Not applicable

**Main outcome(s) and measure(s):**

Characteristics of patients who died by EAS because of suffering from a PD, characteristics of the reporting physician and consultant(s) and the RTEs assessment of published cases.

**Results:**

Of the 72 cases studied, the majority of patients were female (n=48, 67%), suffered from 3 or more conditions (n=38, 53%) and died by euthanasia instead of assistance in suicide (n=56, 78%). In 63% of cases (n=45), the life termination was performed by a physician from the Euthanasia Expertise center (EE). The RTEs’ judgement that the case did not meet the due care criteria (n=11) was in all cases related to issues regarding the (advice of the) independent physician or psychiatric expert.

**Conclusion and relevance:**

This qualitative study shows that the RTEs attach great importance to a careful evaluation procedure of physicians handling EAS requests and to the physician demonstrating ability to reflect on his views, especially when the independent consultant evaluates the case different than the physician. Training for physicians and more transparency in the assessment of EAS requests in patients with a PD may lower the threshold for physicians to handle requests of these patients themselves.

**Trial registration:**

Not applicable.

## Introduction

In the Netherlands, it is possible for physicians to end a patient’s life through either euthanasia, where a physician administers lethal medication to the patient, or assisted suicide, in which case the patient self-administers lethal medication provided by a physician. These procedures are considered legal if the physician complies with the criteria of due care laid down in Article 2 (1) of the Dutch euthanasia act (**Box 1**) (1, 2).

Box 1 **The statutory criteria of due care.**

The physician must:

a) be satisfied that the patient’s request is voluntary and well considered.

b) be satisfied that the patient’s suffering is unbearable, with no prospect of improvement.

c) have informed the patient about his situation and his prognosis.

d) have come to the conclusion, together with the patient, that there is no reasonable alternative in the patient’s situation.

e) have consulted at least one other, independent physician, who must see the patient and give a written opinion on whether the due care criteria set out in (a) to (d) have been fulfilled.

f) have exercised due medical care and attention in terminating the patient’s life or assisting in the patient’s suicide.

The law stipulates that each case of euthanasia or assisted suicide (EAS) must be reported to one of five Regional Euthanasia Review Committees (RTEs), which subsequently assesses whether the physician acted in accordance with the due care criteria (3). If the RTEs conclude that the physician did not act in accordance with the due care criteria, they are legally required to report this finding to the Public Prosecution Service and the Health and Youth Care Inspectorate. These bodies will then consider the appropriate next step (4). The due care criteria are formulated broadly. The RTEs provide an explanation of how the criteria should be interpreted in their Euthanasia Code. The code is based on the law, norms developed in the RTEs’ judgements on individual cases, and on case law. Besides clarifying the interpretation of the criteria of due care, the Euthanasia Code also explicates the assessment procedure (4).

One of the conditions for qualifying for EAS, based on case law, is that the patient’s suffering predominantly originates from a medically classifiable disease. Since there is no requirement that the patient’s suffering stems from a somatic disease or a life-threatening condition, patients who suffer from a psychiatric disorder (PD) are not precluded from being eligible for EAS (4).

Despite the legal possibility of EAS in patients suffering from a PD, which is supported by about half of Dutch citizens (55%), most physicians (64%) find it inconceivable to ever perform EAS in patients suffering from a PD (5, 6). It is seen as a complex matter requiring diligence and specific expertise (7). Therefore, in addition to the standard statutory criteria of due care, the RTEs expect the physician to exercise “extra caution” in evaluating a request for EAS by a patient suffering from a PD (4). Such extra caution relates, for example, to the criterion on decisional capacity. In general, the Euthanasia Code states that the patient “must fulfil four criteria: the patient must 1) be able to communicate intelligibly about their request, 2) be able to understand the relevant medical and other information about their situation and prognosis, 3) have insight into their condition, and 4) be able to make clear why they want euthanasia to be performed” (p.19). For requests of patients suffering from a PD the code adds: “The physician must rule out that the patient’s competence is impaired by their psychiatric disorder. The physician must take particular note of whether the patient is able to grasp relevant information, understands their disease and is unequivocal in their request” (p.44). Further details on the RTEs’ interpretation of the due care criteria in PD cases are summarized in **Box 2** (4).

Box 2 **Extra caution required in evaluating PD cases (bulleting is according to criteria a-f in Box 1).**

According to the RTEs:

a) The physician must rule out that the patient’s competence is impaired by their psychiatric disorder. The physician must take particular note of whether the patient is able to grasp relevant information, understands their disease and is unequivocal in their request.

b) The physician must carefully explore the possibility of other options that could end or reduce the patient’s suffering (especially when the patient’s life expectancy is relatively long).

d) If the patient refuses a reasonable alternative, they cannot be said to be suffering with no prospect of improvement. At the same time, patients are not obliged to undergo every conceivable form of treatment or intervention.

a,b,d) The physician must seek psychiatric expertise. An independent psychiatrist must assess.

▪ The patient’s decisional competence with regard to their request for EAS.

▪ Whether the patient is suffering unbearably.

▪ Whether there are no reasonable alternatives.

e) The physician is free to choose whether he will consult an independent psychiatrist in addition to an independent (SCEN^a^) physician, or an independent (SCEN) physician who is also a psychiatrist.

a SCEN stands for Support and Consultation on Euthanasia in the Netherlands. A SCEN physician is a trained, independent expert who provides the physician with support, information and formal consultation during an EAS trajectory.

To date EAS in patients suffering from a PD is only possible in a few countries, besides the Netherlands, including Belgium, Luxembourg and Switzerland (only assistance in suicide) (8). The practice is seen as highly controversial in most countries (9).

From 2008, cases of EAS in a patient with a PD are specified in the RTEs annual reports (**Table 1**). Since then, there has been an increase in reported PD cases, leading up to a total of 138 cases in 2023 (1.52% of all reported EAS cases).

**Table 1:** The total number of annually reported cases of EAS^1^ and the number of PD cases, from 2008 to 2023 (Regional Euthanasia Review Committees, 2008-2023).

Year	Reported cases (total)	Reported PD cases	Share PD cases	PD cases handled by an EE physician
	N	N	%	N (%)
2008	2.331	2	0,09%	–
2009	2.636	0	0,00%	–
2010	3.136	2	0,06%	–
2011	3.695	13	0,35%	–
2012	4.188	14	0,33%	–
2013	4.829	42	0,87%	–
2014	5.306	41	0,77%	–
2015	5.516	56	1,02%	–
2016	6.091	60	0,99%	37 (62)
2017	6.585	83	1,26%	52 (63)
2018	6.126	67	1,09%	44 (66)
2019	6.361	68	1,07%	52 (76)
2020	6.938	88	1,27%	68 (77)
2021	7.666	115	1,50%	83 (72)
2022	8.720	115	1,32%	65 (57)
2023	9.068	138	1.52%	70 (51)

- Not specified in annual reports.

^1^EAS, euthanasia or assisted suicide; PD, psychiatric disorder; EE, Euthanasia Expertise center.

It is known that physicians are reluctant to consider or grant a patient’s request for EAS based on suffering from a PD (5, 9–11). When physicians are unable or unwilling to handle an EAS request, they can seek help from, or refer the patient to, the Euthanasia Expertise center (EE). The EE was founded in 2012. Currently, a large proportion of EAS requests from patients suffering from a PD are not being handled by their own physician, but by an EE physician instead. This has resulted in waiting times of up to two years before an EAS request of a patient suffering from a PD can be considered by the EE (12). In the period 2017-2022, 10% of all incoming EAS requests from patients suffering from a PD were granted by the EE (13).

The RTEs publish all summarized and anonymized cases that were assessed as not in accordance with the criteria of due care on their website, as well as a selection of the remaining cases – assessed as in accordance with the criteria of due care – that are “important for developing norms or for societal debate” (14, 15). Previous research focused on published cases that were not in compliance with the criteria of due care (16), and on cases of EAS in patients with autism (17), multiple geriatric syndromes (18), and dementia (19). Kim et al. and Van Veen et al. provided insight in characteristics and psychiatric pathologies underlying the EAS request of patients suffering from a PD between 2011 and 2017 (20, 21), and Nicolini et al. focused on patients with personality disorders (8).

The Netherlands is in a unique position to provide insights into this practice. However, sources that accurately describe the Dutch situation are largely unavailable to international readers, increasing the risk of misunderstandings. Our aim is to contribute to better insight into the Dutch practice of EAS in patients suffering from a PD for an international audience. To this end we analyzed the RTEs assessments of published cases of EAS in patients suffering from a PD between 2017 and 2022, and in particular the RTEs’ considerations in the cases in which the physician did not act in accordance with the due care criteria.

## Methods

### Study design, case extraction and analysis

We performed a retrospective case review study in which we extracted all published cases of EAS in patients suffering from a PD between 2017 and 2022 from the RTEs’ website. Inclusion criteria were:

- Cases labelled by the RTEs as euthanasia based on “psychiatric disorders”, between 2017 and 2022,.

Exclusion criteria were:

- Main cause of suffering leading to request is not psychiatric suffering.

The RTEs identified 75 cases, but three cases were excluded. In these cases the reason of the EAS request was primarily suffering caused by a somatic disease. The psychological suffering of these patients did not play a significant role in the request for EAS, nor in the RTEs’ assessment of the case. The final number of included cases was thus 72.

The 75 cases were selected for publication by the RTEs from the total of 536 reported cases of EAS in patients with a PD in this period, resulting in a publication rate of 14% (13). For each case, the following characteristics were identified and independently mapped by researchers FB and KRM: year of death, sex and age of the patient, specialization of the notifying physician, number of consulted independent physicians and their specializations, the type of life termination (euthanasia, assisted suicide or a combination of both), the number of conditions from which the patient suffered, and whether these conditions were solely psychiatric or a combination of psychiatric and somatic disorders. Data that yielded questions or were unclear were discussed by all authors. Case characteristics were analyzed and summarized using Excel statistics.

We further analyzed 11 cases in which the RTEs assessed that the physician did not act in accordance with the due care criteria, with a focus on the required extra caution. FB extracted information from the case descriptions which was checked by KM. The full research team read the extracts to judge whether they were complete, coherent and understandable. Case characteristics were analyzed and summarized using Excel statistics.

This article follows the Standards for Reporting Qualitative Research (SRQR) guidelines (22).

## Results

### Characteristics of published PD cases

**Table 2** shows an overview of characteristics of cases of EAS in patients with a PD between 2017 and 2022. The majority of patients were female (n=48, 67%), under the age of 80 (n=62, 86%), and died by euthanasia instead of assistance in suicide (n=56, 78%). Most patients suffered solely from a psychiatric disorder (n=48, 67%), and often more than one disorder. In 63% of cases (n=45), the life termination was performed by a physician from the EE.

**Table 2:** Characteristics of published cases of EAS in patients with a PD between 2017 and 2022^a^.

	Cases ‘in accordance with due care criteria’ (N=61) N (%)^b^	Cases ‘not in accordance with due care criteria’ (N=11) N (%)^b^	Total cases (N=72) N (%)^b^
Year			
2017	7 (11)	1 (9)	8 (11)
2018	9 (15)	2 (18)	11 (15)
2019	17 (28)	1 (9)	18 (25)
2020	14 (23)	0 (0)	14 (19)
2021	8 (13)	3 (27)	11 (15)
2022	6 (10)	4 (36)	10 (14)
Sex			
Female	42 (69)	6 (55)	48 (67)
Male	19 (31)	5 (45)	24 (33)
Age			
18-30	8 (13)	0 (0)	8 (11)
30-60	26 (43)	5 (45)	31 (43)
60-80	17 (28)	6 (55)	23 (32)
>80	10 (16)	0 (0)	10 (14)
Type of condition			
Solely psychiatric disorder(s)	41 (67)	7 (64)	48 (67)
Combination of psychiatric and somatic disorders	20 (33)	4 (36)	24 (33)
Type of life termination			
Euthanasia	46 (75)	10 (91)	56 (78)
Assisted suicide	8 (13)	1 (13)	9 (13)
Combination^c^	7 (11)	0 (0)	7 (10)
Type of physician			
EE physician			
Psychiatrist	28 (46)	3 (27)	31 (43)
Other	12 (20)	2 (18)	14 (19)
Non-EE physician			
Psychiatrist	8 (13)	0 (0)	8 (11)
Other	13 (21)	6 (55)	19 (26)
Number of independent physicians consulted			
1	0 (0)	1 (9)	1 (1)
2	48 (79)	10 (9)	58 (80)
≥ 3	13 (21)	0 (0)	13 (18)

a EAS, euthanasia or assisted suicide; PD, psychiatric disorder; EE, Euthanasia Expertise center.

b Because of rounding, percentages do not always add up to 100 per cent.

c A combination of assisted suicide and euthanasia may occur when the self-administered lethal barbiturate does not lead to a coma and/or death and the physician then administers the barbiturate intravenously (Regional Review Committees, 2022).

In 15% (n=11) of the published cases, the physician’s act was assessed by the RTEs as ‘not in accordance with the due care criteria’ (**Table 2**). The percentage of the total of 536 PD cases that was assessed as not being in accordance with the due care criteria during 2017-2022 was much lower: 1.3% (13). In the published cases the male-female distribution was almost equal (n=5, 45% and n=6, 55% respectively), as well as the distribution of physicians who were and were not associated with the EE (n=6, 55% and n=5, 45% respectively). In 91% of cases (n=10) the patient died by euthanasia.

### Cases not in accordance with due care criteria

#### Independent consultant was not a psychiatrist

In 7 cases (2017-24; 2021-143; 2021-97; 2021-76; 2022-039; 2022-075; 2022-068), the physician consulted an independent physician who was not a psychiatrist, and failed to display the additional required caution of consulting at least one psychiatrist to evaluate criteria a, b and d.

#### Independent consulted psychiatrist did not evaluate all criteria

In another case (2019-15) the physician had consulted two independent psychiatrists to evaluate criteria a, b and d, as the required extra caution prescribes, but failed to also ask for an evaluation of criterion c, which is part of the standard evaluation of an EAS request (see criterion e, **Box 1**). In this case, the physician was aware that he needed to ask an additional independent physician to assess all criteria, but chose not to do so because another consultation would have been “very burdensome” for the patient, and because he believed that he had already been very careful in evaluating the patients’ request. In the physician’s opinion, another consultation would not add to a careful evaluation, but merely represent “a formal completion of the procedure”. The RTEs wrote in their assessment: “Although he [the physician, FB] had consulted two independent psychiatrists who evaluated the patient’s capacity, hopelessness and treatment options, he had not approached a consultant who gave his assessment on *all* criteria of due care”. Therefore, the physician was considered not to have acted in accordance with the criteria of due care.

#### Overruling the independent consultant’s negative evaluation

In the remaining 3 cases (2018-70; 2018-69; 2022-017), the notifying physicians (all psychiatrists) were considered not to have exercised sufficient caution because they overruled the negative evaluation of the consulted independent physician(s) (also psychiatrists) regarding criteria a, b and d, and did so with “insufficient arguments”. The RTEs wrote in their assessment of these cases that in evaluating an EAS request from a patient suffering from a PD, a negative evaluation by a consulted physician carries “even more weight”.

In the first case (2018-69) two psychiatrists were consulted. The first consultant was not convinced of the patient’s unbearable suffering, but the report about this finding was “extremely brief” and “did not demonstrate thorough investigation”. Therefore, the RTEs considered this consultation inadequate. The second consultant, an experienced psychiatrist, doubted not only that the patient’s unbearable suffering was without prospect of relief, but also the lack of reasonable alternatives to alleviate the patient’s suffering. The physician indicated that he saw no reason to consult another independent psychiatrist, because the negative evaluation of the former consultants did not make the physician doubt the patient’s suffering. Furthermore, the physician argued he did not want to burden the patient again, and did not want to create the impression that he was “shopping around” for positive evaluation. The RTEs found these arguments insufficient, and wrote in their assessment: “The report [of the second consultant, FB] clearly and unequivocally argued why euthanasia should not be performed in this case. Nevertheless, the physician stuck to his own view, without further questioning his own assessment. (…) The Committee is of the opinion that in the given circumstances - including the limited report of the first consultant - the physician should certainly have asked another independent consultant, preferably also a psychiatrist, or an independent expert, also to protect himself from possible tunnel vision”.

In the second case (2018-70) the consulted independent psychiatrist noted that at the time of the consultation, the patient’s chronic psychiatric symptoms were in the background, and somatically unexplained symptoms were in the front. He advised to try a specific treatment option with an admittedly low, but not nil chance of success, aimed at reducing the patient’s suffering. The patient attended an intake interview at a specialised programme to see if he suffered from chronic fatigue syndrome that could be treated with cognitive behavioural therapy, but the diagnosis could not be made after one interview. The patient declined the proposal of further diagnostic interviews as he could not bring himself to engage in further treatment. A second independent consultant (not a psychiatrist) advised the physician to seek additional psychiatric advice to evaluate the patient’s suffering, but this was waived by the attending physician. The RTEs were not convinced by the physician’s arguments for overruling the independent consultant’s opinion and advice and state: “(…) Although not a mandatory requirement, the obvious course of action then is to approach another independent physician, preferably also a psychiatrist”.

Lastly, in the third case (2022-017), the psychiatrist that was consulted firstly evaluated the patient as not being competent regarding his EAS request. However, according to the second independent consultant (not a psychiatrist), all due care criteria, including the patient’s competence with regard to the voluntary and well-considered request, could be met. The physician argued that there was a “difference in interpretation” regarding the patient’s competence, but the RTEs wrote in their assessment that the physician was “insufficiently open to the views of other physicians”, which led them to conclude that the criteria of due care were not met.

## Discussion

### Interpretation of the results

Almost two thirds of the patients included in our study who died by EAS based on suffering from a PD were under 80 years of age, suffered from more than one psychiatric disorder, and were female. Previous studies also found an overrepresentation of females (70% and 77%) (20, 21). This differs from the equal male-female distribution in EAS cases in general (50.6% and 49.4% respectively in 2022) (23). A possible explanation for this overrepresentation might be that, in general, women are more likely to develop a psychiatric disorder than men, especially depression or anxiety disorders (24). Kim et al. (2016) and Van Veen et al. (2018) showed that these disorders are most common among patients suffering from a PD requesting EAS.

Most of the studied cases were handled by a physician associated with the EE. This reflects the findings from previous research that the majority of physicians are reluctant to consider or grant requests of patients suffering from a PD (5, 9–11). However, a shift occurred in 2023, where there was a decrease in reports of EAS in patients suffering from a PD performed by EE physicians (25). Still, given the long waiting times at the EE before a patient’s EAS request based on suffering from a PD can be addressed, referring all these patients is not desirable (12).

In the majority of cases, life was ended by means of euthanasia instead of assistance in suicide. This reflects a general reluctance in the Dutch practice of physician-assisted dying, where euthanasia is preferred over assisted suicide in the large majority of cases (6).

Furthermore, our study shows that in all cases assessed as not in accordance with the due care criteria, this assessment was related to issues regarding the (advice of the) independent physician. In the majority of these cases the physician consulted 2 independent physicians in evaluating the EAS request, but these consultants were not psychiatrists. This indicates a possible lack of knowledge amongst physicians regarding the extra caution that is required in evaluating an EAS request from a patient suffering from a PD.

In the cases in which the physician ignored an explicit negative advice from the independent consultant to proceed with EAS, the RTEs attach great importance to the physician’s ability to reflect on his own views and to be open to the advice of colleagues. If a physician deviates from the independent consultant’s negative advice, he must adequately justify his decision. This is not only expected from physicians who evaluate EAS requests in patients with a PD, but concerns *all* EAS requests (4). However, the RTEs seem to expect physicians to substantiate their decision even more strongly in cases of EAS in patients suffering from a PD. Moreover, they state it is an “obvious course of action” to approach another psychiatrist when the first consultant gives a negative advice. Given the fact that some physicians did not see the need to consult more independent colleagues in such cases perhaps means that the Euthanasia Code is not clear enough about this expectation.

## Strengths and limitations

Our study has several strengths. The Dutch system of (reporting) EAS is transparent, making data available and allowing us to study the practice of EAS in general, and in patients suffering from a PD in particular. Furthermore, our study builds upon previous research by Kim et al. (2016) and Van Veen et al. (2018) on case characteristics of patients who received EAS based on suffering from a PD and the RTEs’ assessment of these cases.

There are also some limitations. The cases we studied represent only a small proportion of the total number of assessed PD cases. Only 12% of all PD cases assessed as ‘in accordance with the due care criteria’ were published. In addition, the published cases are revised and edited by the RTEs for the general public. We did not have access to the RTEs’ internal discussions or to the original written reports by the notifying physician and the consulted independent physician(s). Lastly, we cannot provide insight into the entire practice of EAS in patients suffering from a PD, since we have no insight in EAS requests that were rejected or not carried out.

## Conclusion and recommendations

As the annual number of notified cases of EAS in patients suffering from a PD continues to rise, and the complexity of such cases remains unabated, our study aimed to provide further insight into the characteristics and RTEs’ assessment of cases of EAS in patients suffering from a PD. To this end, we described the characteristics of 72 published cases between 2017 and 2022 of EAS in patients suffering from a PD, as well as the RTEs’ assessment of cases in which the physician did not act in accordance with the due care criteria.

We found that the majority of patients suffering from a PD who died by EAS were female and died by euthanasia. In almost two thirds of cases the life termination was performed by a physician from the EE. The RTEs’ judgement that the case did not meet the due care criteria was in all cases related to issues regarding the (advice of). the independent physician and how physicians responded to their deviating views.

Given the reluctance of physicians to consider or grant EAS requests of patients with a PD, leading to current long waiting times at the EE, adequate training for physicians to evaluate such EAS requests may lower the threshold for physicians to handle requests of these patients themselves. Such training should be rooted in a substantive professional debate on crucial issues in this practice, such as the assessment of decisional capacity and death wish among patients suffering from PD, as well as research among potentially vulnerable groups such as young women and older people.

## Data Availability

The raw data supporting the conclusions of this article will be made available by the authors, without undue reservation.

## References

[1] Termination of life on request and assisted suicide (review procedures) Act. (2002). Available online at: https://wetten.overheid.nl/BWBR0012410/2021-10-01 (Accessed May 13, 2024). 10.2143/ep.9.2.503855 15712446

[2] Criminal code, articles 293-294. Available online at: https://wetten.overheid.nl/jci1.3:c:BWBR0001854 (Accessed May 13, 2024).

[3] Criminal code, article 293.

[4] Regional Euthanasia Review Committees. Euthanasia Code 2022. Review procedures in practice. Utrecht: Cross Media (2022).

[5] Bolt EE Snijdewind MC Willems DL van der Heide A Onwuteaka-Philipsen BD. Can physicians conceive of performing euthanasia in case of psychiatric disease, dementia or being tired of living? J Med Ethics. (2015) 41:592–8. doi: 10.1136/medethics-2014-102150 25693947

[6] Van der Heide A Onwuteaka-Philipsen B Legemaate J Bosma F Van Delden H Mevis P. Fourth evaluation of the termination of life and request and assisted suicide act. The Hague: Schultenprint (2023).

[7] NVvP Dutch Association for Psychiatry. Richtlijn levensbeeindiging op verzoek bij patienten met een psychische stoornis [Guideline termination of life on request in patients with a psychiatric disorder]. (2018).

[8] Nicolini ME Kim SYH Churchill ME Gastmans C. Should euthanasia and assisted suicide for psychiatric disorders be permitted? A systematic review of reasons. Psychol Med. (2020) 50:1241–56. doi: 10.1017/S0033291720001543 32482180

[9] van Veen S Widdershoven G Beekman A Evans N. Physician assisted death for psychiatric suffering: experiences in the Netherlands. Front Psychiatry. (2022) 13:895387. doi: 10.3389/fpsyt.2022.895387 35795029 PMC9251055

[10] Onwuteaka-Philipsen B Legemaate J van der Heide A Van Delden J Evenblij K El Hammoud I. Third Evaluation of the termination of life and request and assisted suicide act. The Hague: Schultenprint (2017).

[11] Pronk R Evenblij K Willems DL van de Vathorst S. Considerations by dutch psychiatrists regarding euthanasia and physician-assisted suicide in psychiatry: A qualitative study. J Clin Psychiatry. (2019) 80. doi: 10.4088/JCP.19m12736 31721484

[12] Euthanasia Expertise center. Annual report 2023. The Hague: Euthanasia Expertise center. (2024)

[13] Euthanasia Expertise center. Annual reports 2017-2022. The Hague: Euthanasia Expertise center.

[14] Regional Euthanasia Review Committees. Information for researchers . Available online at: https://www.euthanasiecommissie.nl/de-toetsingscommissies/woordvoering-en-voorlichting/informatie-voor-onderzoekers. (Accessed April 15, 2024).

[15] Regional Euthanasia Review Committees. Alle uitspraken en uitleg. Available online at: https://www.euthanasiecommissie.nl/uitspraken-en-uitleg. (Accessed April 15, 2024).

[16] Miller DG Kim SYH. Euthanasia and physician-assisted suicide not meeting due care criteria in the Netherlands: a qualitative review of review committee judgements. BMJ Open. (2017) 7:e017628. doi: 10.1136/bmjopen-2017-017628 PMC5665211 29074515

[17] Tuffrey-Wijne I Curfs L Finlay I Hollins S. Euthanasia and assisted suicide for people with an intellectual disability and/or autism spectrum disorder: an examination of nine relevant euthanasia cases in the Netherlands (2012-2016). BMC Med Ethics. (2018) 19:17. doi: 10.1186/s12910-018-0257-6 29506512 PMC5838868

[18] van den Berg V van Thiel G Zomers M Hartog I Leget C Sachs A. Euthanasia and physician-assisted suicide in patients with multiple geriatric syndromes. JAMA Intern Med. (2021) 181:245–50. doi: 10.1001/jamainternmed.2020.6895 PMC7851730 33284324

[19] Groenewoud AS Leijten E van den Oever S van Sommeren J Boer TA. The ethics of euthanasia in dementia: A qualitative content analysis of case summaries (2012-2020). J Am Geriatr Soc. (2022) 70:1704–16. doi: 10.1111/jgs.17707 PMC9306787 35187649

[20] Kim SY De Vries RG Peteet JR. Euthanasia and assisted suicide of patients with psychiatric disorders in the Netherlands 2011 to 2014. JAMA Psychiatry. (2016) 73:362–8. doi: 10.1001/jamapsychiatry.2015.2887 PMC5530592 26864709

[21] van Veen SMP Weerheim FW Mostert M van Delden JJM. Euthanasia of Dutch psychiatric patients in 2015-2017. Tijdschr Psychiatr. (2019) 61:241–7. 31017282

[22] O'Brien BC Harris IB Beckman TJ Reed DA Cook DA. Standards for reporting qualitative research: a synthesis of recommendations. Acad Med. (2014) 89:1245–51. doi: 10.1097/ACM.0000000000000388 24979285

[23] Regional Euthanasia Review Committees. Annual report 2022. The Hague (the Netherlands: De Bink/OBT. (2023).

[24] Ten Have M Tuithof M van Dorsselaer S Schouten F Luik AI de Graaf R. Prevalence and trends of common mental disorders from 2007-2009 to 2019-2022: results from the Netherlands Mental Health Survey and Incidence Studies (NEMESIS), including comparison of prevalence rates before vs. during the COVID-19 pandemic. World Psychiatry. (2023) 22:275–85. doi: 10.1002/wps.21087 PMC10168151 37159351

[25] Regional Euthanasia Review Committees. Annual report 2023. Regional Euthanasia Review Committees (2022)

